# *Alpinia oxyphylla* Miq. extract changes miRNA expression profiles in db-/db- mouse kidney

**DOI:** 10.1186/s40659-017-0111-1

**Published:** 2017-03-01

**Authors:** Guankui Du, Man Xiao, Xuezi Zhang, Maoyu Wen, Chi Pang, Shangfei Jiang, Shenggang Sang, Yiqiang Xie

**Affiliations:** 1 Department of Biochemistry and Molecular Biology, Hainan Medical College, Haikou, 571101 China; 2 Affiliated Hospital of Hainan Medical College, Haikou, 571199 China; 3 College of Chinese Traditional Medicine, Hainan Medical College, Haikou, 571199 China; 4 Clinical Laboratory, Hainan Medical College Affiliated Hospital, Haikou, 571199 China

**Keywords:** miRNA, db-/db- mice, *Alpinia oxyphylla* Miq, Kidney, Diabetic nephropathy

## Abstract

**Background:**

A number of dysregulated miRNAs have been identified and are proposed to have significant roles in the pathogenesis of type 2 diabetes mellitus or renal pathology. *Alpinia oxyphylla* has shown significant anti-inflammatory properties and play an anti-diabetes role. The objective of this study was to detect the alteration of miRNAs underlying the anti-diabetes effects of *A. oxyphylla* extract (AOE) in a type II diabetic animal model (C57BIKsj db-/db-).

**Results:**

Treatment with AOE for 8 weeks led to lower concentrations of blood glucose, urine albumin, and urine creatinine. 17 and 13 miRNAs were statistically identified as differentially regulated in the DB/DB and db-/db- AOE mice, respectively, compared to the untreated db-/db- mice. Of these, 7 miRNAs were identified in both comparison groups, and these 7 miRNAs were verified by quantitative real-time PCR. Functional bioinformatics showed that the putative target genes of 7 miRNAs were associated with several diabetes effects and signaling pathways.

**Conclusions:**

These founding suggest that the potential of AOE as a medicinal anti-diabetes treatment through changes in the expressions of specific miRNAs. The results provide a useful resource for future investigation of the role of AOE-regulated miRNAs in diabetes mellitus.

**Electronic supplementary material:**

The online version of this article (doi:10.1186/s40659-017-0111-1) contains supplementary material, which is available to authorized users.

## Background

The incidence of diabetes mellitus (DM) is projected to rise to 439 million by 2030, making it among the most important public health challenges [1]. Diabetic nephropathy (DN), diabetes with albuminuria and/or impaired glomerular filtration rate [2], results in one-third of all type 2 DM (T2DM) patients and is the single most important cause of end-stage renal disease [3, 4]. Inflammation appears to be the final common pathway in the development and progression of renal fibrosis [5, 6]. The db-/db- mutant mouse is a rodent model of genetic diabetes that develops renal glomerular lesions with striking mesangial matrix accumulation by the age of 16 weeks after 8–10 weeks of sustained hyperglycemia [7].

miRNAs are small non-coding regulatory RNAs (20–22 nucleotides) that play a key role in regulating numerous biological processes, as well as in the pathology of diseases [8]. In previous studies, miRNA expression profiling was often performed, including determining the circulating miRNAs in DM patients or the miRNAs in different animal-model tissues [9–11]. A number of dysregulated miRNAs were identified and are proposed to have significant roles in the pathogenesis of T2DM or renal pathology [11, 12]. Recent studies have shown that several miRNAs can promote the accumulation of extracellular matrix proteins related to fibrosis and glomerular dysfunction [13]; however, few miRNAs might actually be exploited as biomarkers for the early detection of or new therapeutic targets to prevent the progression of DN.

*Alpinia oxyphylla* (*A. oxyphylla*) is regarded as a precious drug that is widely distributed in South China. Its fruits are used in Traditional Chinese Medicine for the treatment of intestinal disorders, diarrhea, abdominal pain, dementia, inflammatory conditions, and cancer [14–16]. *A. oxyphylla* is rich in sesquiterpenes, diterpenes, flavonoids, and diarylheptanoids. Pungent diarylheptanoids from *A. oxyphylla* show anti-inflammatory properties [17] and *A. oxyphylla* induced apoptosis and suppressed growth of HepG2 cells might be accomplished through the reactive oxygen species mediated signaling pathway [16]. A few studies have shown that *A. oxyphylla* can promote the migration and proliferation of human adipose tissue-derived stromal cells [18, 19]. In our previous study, we found that *A. oxyphylla* extract (AOE) exhibits antioxidant and anti-diabetes properties [20]; however, the molecular mechanisms underlying the AOE mediated anti-diabetes effects are not well understood. In addition, given the importance of miRNAs in the development of diabetes and obesity, we investigated whether miRNAs play a role in the effects of AOE treatment for DN. In the present study, we investigated miRNA expression profiles using deep sequencing in the kidneys of normal DB/DB mice, and in db-/db- mice treated or untreated with AOE.

## Methods

### Preparation of the plant extract

The ripe fruit of *A. oxyphylla* were purchased from a market specializing in herbs (Haikou, Herb Market, China) in Jan of 2015. The plant was authenticated by Dr. Qiang Liu of the Department of Pharmacognosy, Hainan Medical College, Haikou, China. *A. oxyphylla* was extracted with 640 ml of water for 16 h at 90 °C, two times. The water extract was then lyophilized and stored at room temperature until use. The dry yield was 8% (w/w). The dry powder was dissolved directly by water to proper concentration.

### Animals

In this study, we strictly obeyed the animal protocols approved by the Ethics Committee of Hainan Medical College for Animal Care and Use. For the care and use of animals utilized in this research, we monitored the animals twice per week, and none of animals showed severe ill, died or moribund during the whole experiments.

A total of 24, 3–4 week-old male mice, including 8 DB/DB mice and 16 db-/db- (the mice carry a mutation in the leptin receptor gene) mice on a C57BL/Ks background, were obtained from the Model Animal Research Center of Nanjing University, China. All mice were allowed to acclimatize for 1 week before the 8 week experimental period. The mice were divided into 3 groups with 8 animals in each group. DB/DB mice group and db-/db-H_2_O group were administered placebo (saline) only, db-/db-AOE group was administered with 500 mg/kg of AOE via the intragastric route once a day for 8 weeks (approximately, 0.2 ml in volume).

At the end of the 8-week period, individual mice were placed in metabolic cages to obtain 24-h urine collections. Then, the mice were euthanized under chloral hydrate anesthesia, and blood and kidney samples were collected for analysis. Blood samples were collected from the hepatic portal vein into a tube for EDTA anticoagulation and centrifuged (3000 rpm for 15 min at 4 °C) for separating the plasma. The plasma was then frozen at −70 °C for biochemical analysis. The kidney were excised, weighed and homogenized in a 3:1 v/w of 0.25 M sucrose, 10 mM HEPES, 1 mM EDTA (pH 7.5) buffer. Samples were homogenized for 30 s at 6.45 m/s in an Omni Bead Ruptor (OMNI International IM, GA, USA). The protein concentration in each sample was determined using Bradford protein assay kit (TIANGEN Biotech, Beijing).

#### Measurement of concentration of glucose, albumin and creatinine

These parameters were measured using commercial kits (Jian Cheng Biotechnology Company, Nanjing, China), according to the manufacturer’s instructions.

### RNA isolation

Total miRNA was extracted from mice kidney using the mirVana miRNA Isolation kit (Applied Biosystems, USA) according to the manufacturer’s instructions.

### Sequencing and reads processing

For small-RNA sequencing, complementary small-RNA libraries were prepared by ligating different adaptors to the total RNA followed by reverse transcription and polymerase chain reaction (PCR) amplification. Sequencing was performed using the Illumina HiSeq 2000 sequencer (Illumina, USA) with 50-bp single-end reads according to the manufacturer’s standard protocol. The removal of poor quality sequences and trimming of adaptor sequences from the raw sequence data was carried out using cutadapt [21], trimmed sequences shorter than 18 nt was discarded. The clean sequencing data were mapped to the mouse genome (release GRCm37.p1, from NCBI genome database) and Rfam database v11 (http://www.sanger.ac.uk/Software/Rfm/). Reads aligned in the genome, excluding those matching tRNAs, rRNAs, snRNA, and snoRNAs, were used for further analysis. All known mature miRNAs and their precursors were retrieved from miRBase (version 21; http://www.mirbase.org).

### miRNA identification and qualification

The remaining reads were used to predict novel miRNAs and do quantitative analysis through the miRDeep2 [22]. The frequency of microRNAs from different libraries was normalized by total clean reads of microRNAs in each sample. If the normalized read count of a given microRNA is zero, the expression value was modified to 1 for further analysis. The pairwise t test was applied to filter differentially expressed microRNAs and mRNAs for the two groups. For each miRNA,reads number was normalized. False discovery rate (FDR)—adjusted P values (P 0.05) and an absolute fold change of 1 were set as the cutoff values.

### Hierarchical clustering

Hierarchical clustering was applied to both axes using the weighted pair-group method with centroid average as implemented in the program Cluster (Eisen; http://www.microarrays.org/software). The distance matrixes used were Pearson correlation for clustering the arrays and the inner product of vectors normalized to magnitude 1 for the genes (this is a slight variant of Pearson correlation; see Cluster manual available at http://www.microarrays.org/software/ for computational details). The results were analyzed with Tree View (Eisen; http://www.microarrays.org/software) [23].

### Validation of differentially expressed miRNAs

Quantitative real time (qRT)-PCR was performed to confirm the differential expression of miRNAs identified by sequencing. Briefly, cDNA synthesis and qRT-PCR were performed using TaqMan miRNA assays (Applied Biosystems, Foster City, CA, USA) according to the manufacturer’s instructions. Cycle threshold (Ct) values for miRNAs were normalized against U6 RNA (internal control) and the relative expression was calculated using the 2^−ΔΔCt^ method.

### Predication of the potential target miRNAs

There is no one algorithm that outperforms the others in terms of sensitivity and specificity. The potential miRNAs target genes were identified by miRWalk, miRanda, Sanger miRDB, RNAhybrid, and Targetscan in the most commonly used prediction website (http://www.umm.uni-heidelberg.de/apps/zmf/mirwalk/predictedMiRNAsgene.html) [24]. Gene function was assigned based on Database for Annotation, Visualization and Integrated Discovery (DAVID).

### Statistical analyses

The concentration of glucose, albumin and creatinine are presented as the mean ± standard deviation (SD). Data were analyzed by the Statistical Product and Service Solutions (SPSS) program (Version 16) (IBM, USA). Comparisons of multiple groups were done with ANOVA with corrections for multiple comparisons. Differences of P < 0.05 were considered statistically significant.

## Results

**Fig. 1:**
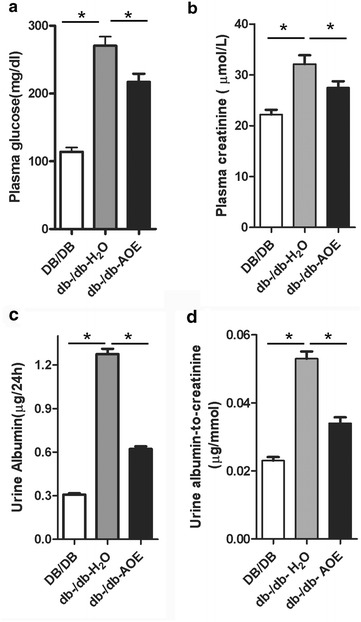
Effects of AOE on blood glucose levels (**a**), plasma creatinine (**b**), urine albumin (**c**) and urine albumin to creatinine (**d**). Data represent the mean ± SD (n = 8). *P < 0.05

Changes in body weight of db-/db- mice after AOE administration for 8 weeks did not differ (data not shown). In db-/db-AOE mice, plasma glucose decreased significantly by 28% (P < 0.001), plasma creatinine (Cr) decreased significantly by 16.7% (P < 0.05), urine albumin excretion decreased by 52% (P < 0.001), and urine albumin-to-creatinine levels decreased 35.9% (P < 0.001), comparing with db-/db-H_2_O group (Fig. 1). Meanwhile, all parameters we evaluated in db-/db-AOE group were higher than DB/DB group. Those results suggest that AOE plays a role in both anti-diabetes and improving renal function.

**Fig. 2:**
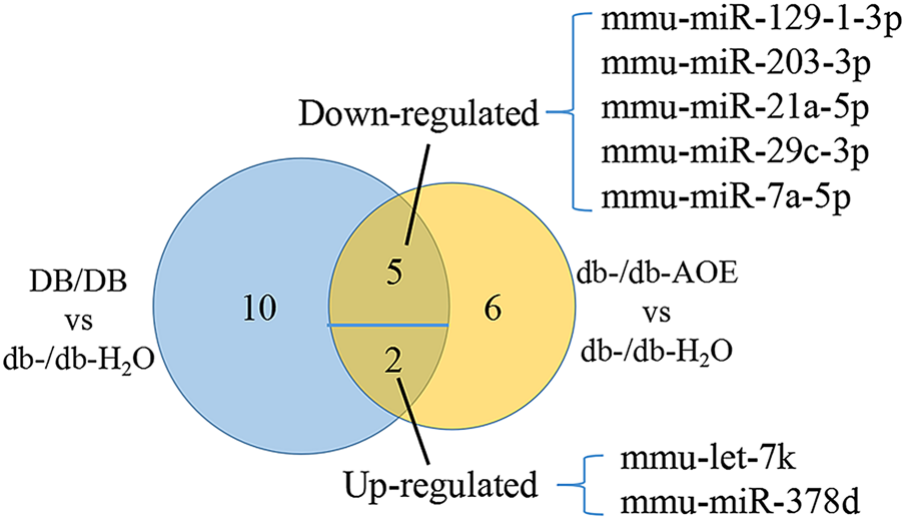
*Venn diagrams* showing the overlap between DB/DB group vs db-/db-H_2_O group and db-/db-AOE group vs db-/db-H_2_O group

**Fig. 3:**
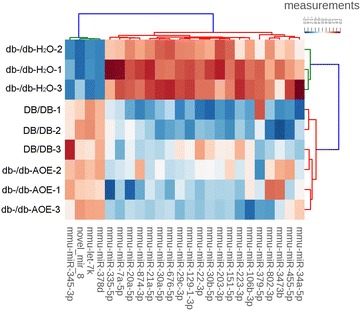
Hierarchical clustering of kidney tissues from DB/DB mice and db-/db- mice treated and untreated with AOE. Kidney tissue was clustered according to the expression profiles of 23 differentially expressed miRNAs between db-/db- and DB/DB groups and db-/db- mice treated and untreated with AOE. The analyzed samples are reported in columns and the miRNAs are presented in rows. The miRNA dendrogram is shown on the left, and the sample dendrogram appears at the *top*. The *color scale* shown at the *top* indicates the relative expression level of miRNAs, with *red* representing a high expression level and *blue* representing a low expression level

Then, we tried to more deeply understand the molecular mechanisms of AOE therapy for DN. miRNA expression patterns in the kidneys of DB/DB and db-/db- mice were detected by using deep sequencing and differential expression analysis. We compared the known miRNAs expression between the two samples to determine the differentially expressed miRNAs. In total, 23 miRNAs were significantly (at least two fold) identified as differentially regulated in the DB/DB vs db-/db-H_2_O mice (17 miRNAs) and/or db-/db-AOE vs db-/db-H_2_O mice (13 miRNAs) (Table 1). In them, 7 miRNAs (mmu-let-7k, mmu-miR-378d, mmu-miR-129-1-3p, mmu-miR-21a-5p, mmu-miR-29c-3p, mmu-miR-203-3p, and mmu-miR-7a-5p) were identified in both comparison groups (Fig. 2). All 23 miRNAs were used for hierarchical clustering analysis (Fig. 3). 19 miRNAs are either down- or up-regulated in both comparison groups. Therefore, our results showed diabetes mellitus changed the miRNAs expression pattern, which could be partially reverted by AOE treatment.

**Table 1:** All 24 regulated miRNAs in kidney tissues: 24 miRNAs with fold change and adjusted p-values that were found to be differentially regulated in the diabetes mice (DB/DB vs db-/db-H_2_O) or diabetes mice treated with AOE (db-/db-AOE vs db-/db-H_2_O)

	DB/DB versus db-/db-H_2_O (FC Log2)	P value	db-/db-AOE versus db-/db-H_2_O (FC Log2)	P value
*mmu-let-7k*	*2.59*	*0.00097*	*2.47*	*0.00008*
mmu-miR-106b-3p	−1.03	0.01629	−0.92	0.06316
*mmu-miR-129-1-3p*	−*1.27*	*0.01035*	−*1.15*	*0.00049*
mmu-miR-151-5p	−1.17	0.01808	−0.93	0.05481
*mmu-miR-203-3p*	−*1.13*	*0.01778*	−*1.01*	*0.00634*
mmu-miR-20a-5p	−0.81	0.08940	−1.03	0.01215
*mmu-miR-21a-5p*	−*1.54*	*0.00118*	−*1.34*	*0.00098*
mmu-miR-223-3p	−1.40	0.06575	−1.62	0.01640
mmu-miR-22-3p	−0.89	0.10567	−1.14	0.00635
*mmu-miR-29c-3p*	−*1.10*	*0.01878*	−*1.01*	*0.00147*
mmu-miR-30a-5p	−1.22	0.00060	−0.96	0.05265
mmu-miR-30b-5p	−1.06	0.04934	−0.91	0.06124
mmu-miR-335-5p	−0.62	0.13545	−1.08	0.02466
mmu-miR-345-3p	1.03	0.01347	0.79	0.07587
mmu-miR-3473b	−1.09	0.03752	0.16	0.18946
mmu-miR-34a-5p	−1.02	0.04450	0.26	0.06833
*mmu-miR-378d*	*5.25*	*0.00000*	*5.16*	*0.00014*
mmu-miR-379-5p	−0.39	0.08465	−1.01	0.01962
mmu-miR-455-5p	−1.28	0.00542	0.68	0.08325
mmu-miR-676-5p	−1.31	0.05468	−1.01	0.00082
*mmu-miR-7a-5p*	−*1.31*	*0.00964*	−*1.33*	*0.00793*
mmu-miR-802-3p	−1.38	0.00231	0.64	0.07654
mmu-miR-874-3p	−1.60	0.00441	−1.40	0.05649
novel_mir_8	2.08	0.00431	2.25	0.00258

Italic values indicate the miRNAs was further analyzed via qRT-PCR

**Fig. 4:**
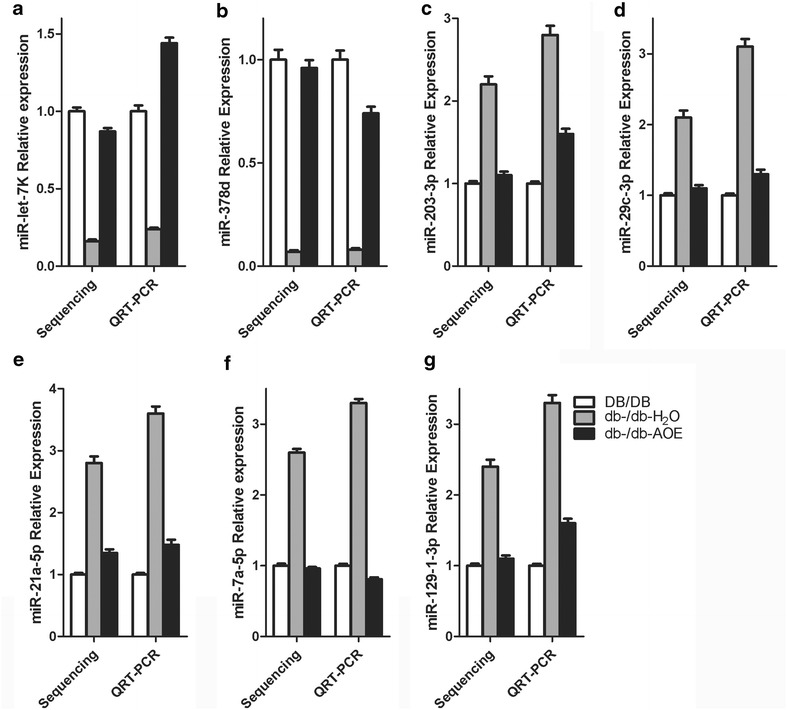
Quantitative real-time polymerase chain reaction (qRT-PCR) validation of the identified miRNAs. The expression of **a** miR-let-7k, **b** miR-129-1-3p, **c** miR-378d, **d** miR-21a-5p, **e** miR-29c-3p, **f** miR-203-3p, **g** miR-7a-5p in DB/DB groups (*white column*), db-/db-H_2_O (*gray column*) and db-/db-AOE (*black column*) detected by QRT-PCR consist with sequencing. Data represent the mean ± SE, The experiment repeated three times

To validate the deep sequencing data, the miRNA expression was measured by qRT-PCR. 7 miRNAs (mmu-let-7k, mmu-miR-378d, mmu-miR-129-1-3p, mmu-miR-21a-5p, mmu-miR-29c-3p, mmu-miR-203-3p, and mmu-miR-7a-5p) were selected as candidate and quantified by qRT-PCR. The qRT-PCR results for miRNAs and mRNAs presented in Fig. 4 demonstrate very good correspondence between the two platforms.

To better understanding the 7 identified miRNAs function, miRWalk was used to predict the miRNA targeted genes. Based on stringent standards, 1071 putative miRNA-target genes were chosen; DAVID tools were also used to search for Kyoto Encyclopedia of Genes and Genomes (KEGG) pathway analysis (Table 2). The results showed that 6 KEGG class pathway was enriched, including Environmental Information Processing, Genetic Information Processing, Human Diseases, Metabolism, Organismal Systems and Environmental Information Processing. Those results suggest AOE might affect diabetes development through miRNAs target genes and related signaling pathway.

**Table 2:** Biologic pathways enriched by differentially expressed microRNAs

KEGG class	KEGG description	Odds ratio	P value	Genes Num
Environmental information processing	*AMPK signaling pathway*	2.22	0.008184	14
	*Jak-STAT signaling pathway*	2.12	0.009269	15
	FoxO signaling pathway	2.09	0.01286	14
	Cell adhesion molecules (CAMs)	2.02	0.013299	15
	ErbB signaling pathway	2.35	0.016766	10
	Cytokine-cytokine receptor interaction	1.63	0.03407	20
	*PI3K-Akt signaling pathway*	1.52	0.036417	26
	ECM-receptor interaction	2.02	0.046688	9
Genetic information processing	Fanconi anemia pathway	2.53	0.042796	6
Human diseases	Choline metabolism in cancer	2.41	0.007808	12
	Pancreatic cancer	2.82	0.008459	9
	Renal cell carcinoma	2.82	0.008459	9
	Glioma	2.50	0.0227	8
	Non-small cell lung cancer	2.59	0.027265	7
	Measles	1.84	0.042725	12
	*Type II diabetes mellitus*	2.53	0.042796	6
	Proteoglycans in cancer	1.65	0.042942	17
Metabolism	Valine, leucine and isoleucine degradation	3.10	0.007801	8
	Lysine degradation	2.83	0.01867	7
	beta-Alanine metabolism	3.41	0.023687	5
	*Glycosphingolipid biosynthesis—lacto and neolacto series*	3.37	0.0429	4
	*Glycosphingolipid biosynthesis—globo series*	4.41	0.043669	3
Organismal systems	Fc epsilon RI signaling pathway	2.67	0.011246	9
	Osteoclast differentiation	1.95	0.031094	12
	Neurotrophin signaling pathway	1.95	0.031094	12
	Cholinergic synapse	2.00	0.032356	11

## Discussion

Diabetic kidney disease is the leading cause of end-stage renal disease. Albuminuria is recognized as the most important prognostic factor for CKD progression [4]. In the present study, we observed that AOE treatment reduced blood glucose levels and urine albumin secretion, while plasma creatinine level and urine albumin to creatinine level was also reduced. Those results suggest that AOE treatment plays a protective role by adjusting renal function.

Kidney abnormalities are associated with aberrant miRNA expression patterns. We assessed the status of miRNA expression by deep sequencing. For small RNA filtration and miRNA annotation, The 49nt sequence tags from Hiseq sequencing gone through the data cleaning analysis to get credible clean tags. A total of 1.18*10^7^ reads were sequenced from the mice small RNA library. Total 10,391,183 (88.93%) clean reads remained after removing ambiguous reads (Additional file 1). After reads assembly, removing the redundancy and annotation of unique sequences, a total of 276,231 Unique sRNAs were obtained, and of them, about 19.45% are the potential miRNA reads with 21–24 bp in length (Additional file 2).

In the kidneys, total 17 miRNAs were statistically identified while db-/db-H_2_O compared with DB/DB. In a recent study, several miRNAs were identified from the renal of db-/db- [11]; however, we did not find any overlap with the miRNAs that were altered in the previous studies when compared with those that we report here. This is presumably because miRNAs exhibit tissue-specific expression patterns. In them, 9 miRNAs (miR-21a, miR-29c, miR-30a, miR-30b, miR-34a, miR-106b, miR-203, miR-378 and miR-802) had been shown to be related with diabetes or glucose metabolism. In diabetic patients, miR-21a is down-regulated in peripheral blood mononuclear cells [25], serum miR-30a and urine miR-30b expression is up-regulated [26, 27]. miR-29c is related with renal interstitial fibrosis in humans and rats [28]. Inhibition of miR-29c significantly reduces albuminuria and kidney mesangial matrix accumulation in the db-/db- mice [29]. Down-regulation of miR-34a alleviates mesangial proliferation in vitro and glomerular hypertrophy in early DN mice [30]. miR-106b is highly expressed in nephron progenitors and negatively regulates insulin sensitivity [31, 32]. miR-203 is modified in diabetic mice, and might responds to hepatic insulin resistance [33, 34]. Overexpression of miR-802 impairs glucose metabolism [35] miR-378 is regulated by glucose concentration, while high level of miR-378 could attenuates high glucose-suppressed osteogenic differentiation in vitro and diabetic mice model [36]. Those miRNAs are related with renal proliferation, interstitial fibrosis, mesangial matrix accumulation or insulin sensitivity, which confirm that we obtain some important miRNAs in DN mice model. Moreover, there is also the first demonstrated elevated levels of miR-874-3p, miR-7a-5p, miR-455-5p, miR-129-1-3p, miR-151-5p, miR-3473b, and down regulated levels of miR-345-3p, novel_mir_8 and let-7 k in the kidneys of db-/db- mice. Our study of the db-/db- mice kidney is a beneficial complement to the current knowledge of the effects of the miRNA expression profile on kidney metabolism during diabetes.

Then, we assume that AOE treatment might change the miRNAs expression pattern in db-/db- mice kidney. Fortunately, we found 13 differential expression miRNAs. In them, 7 miRNAs (miR-378d, miR-29c-3p, miR-20a-5p, miR-335-5p, miR-22-3p, miR-21a-5p and miR-223-3p) had been shown to be related with diabetes or glucose metabolism. In diabetic patients, miR-20a-5p is high expressed [37]. It is reported that miR-22 is involved in renal fibrosis and glucose metabolism [38, 39]. miR-223 and miR-335 are specifically regulated by hyperglycemia, and are crucial regulator of inflammatory response and systemic insulin resistance [40–42]. In total, we found out 23 miRNAs was significantly altered in the DB/DB vs db-/db-H_2_O mice (17 miRNAs) and/or db-/db-AOE vs db-/db-H_2_O mice (13 miRNAs). The alteration of 19 miRNAs showed the similar tendency. We also found the 4 miRNAs expression is oppositely regulated, but the p value suggests it is non-significantly difference. Interestingly, 7 miRNAs (let-7k, miR-378d, miR-129-1-3p, miR-21a-5p, miR-29c-3p, miR-203-3p, and miR-7a-5p) expression was significantly restored after AOE treatment. In them, 4 miRNAs (miR-378d, miR-21a-5p, miR-29c-3p and miR-203-3p) had been shown to be related with renal interstitial fibrosis or glucose metabolism. Thus, we deduced that those 7 miRNAs might act a more authentic role in AOE anti-diabetic therapy.

Furthermore, the target genes regulated by the 7 miRNAs identified were subjected to KEGG pathway enrichment. Our study showed that T2DM; renal cell carcinoma; AMPK signaling pathway; PI3K-Akt signaling pathway; glycosphingolipid biosynthesis; and the Jak-STAT signaling pathway are affected. It is reported that the PI3K-Akt signaling pathway plays a role in insulin-mediated glucose uptake in both muscle and adipose tissue cells while inhibiting glucose release from hepatocytes [43]. AMPK signaling pathway plays an important role in glucose metabolism [44]. Glycosphingolipid synthesis is involved in insulin sensitivity and glucose homeostasis [45]. High levels of glycosphingolipids contribute to cell fibrosis, and causing early diabetic kidney disease [46]. Activation of the JAK/STAT signaling pathway can stimulate unwarranted proliferation and growth of glomerular mesangial cells, resulting in DN [47]. Overall, our KEGG analysis results reveal miRNAs related to DN development and AOE treatment mechanism. Further study will focus on experimental validation of miRNAs of interest and their target genes and pathways.

## Conclusions

We identified 17 different expressions of miRNAs in DB/DB mice vs db-/db- mice and 13 different expressions of miRNAs in db-/db- mice treated vs untreated with AOE. Most of miRNAs that relate to renal failure or T2DM had already been reported. 2 miRNAs were inhibited in db-/db- mice and restored by AOE treatment, while 5 miRNAs were enhanced in db-/db- mice and impaired by AOE treatment. The 7 identified miRNAs might be involved in several pathways, including T2DM, renal cell carcinoma, AMPK signaling pathway, and PI3K-Akt signaling pathway; however, the detailed function associated with these miRNAs in AOE therapy needs further investigation and the target genes of miRNAs need further validation through additional studies.

## Additional files

**Additional file 1.** Overview of mouse transcriptome sequencing reads.

**Additional file 2.** The sequence length distribution and frequence percentage of small RNA reads of mouse. The x-axis indicates the length of small RNA reads. The y-axis indicates the percentage of small RNA reads with specific length.

## Abbreviations

CKD: chronic kidney disease
Ct: cycle threshold
Cr: creatinine
DM: diabetes mellitus
DN: diabetic nephropathy
FDR: false discovery rate
GO: Gene Ontology
KEGG: Kyoto Encyclopedia of Genes and Genomes
mRNAs: messenger RNAs
NCBI: National Center for Biotechnology Information
PCR: polymerase chain reaction
qRT-PCR: quantitative real-time polymerase chain reaction
SD: standard deviation
SPSS: Statistical Product and Service Solutions
T2DM: type 2 diabetes mellitus
