# Supplementary figures and images for: Alpinia oxyphylla Miq. extract changes miRNA expression profiles in db-/db- mouse kidney
Source: Biol Res. 2017 Mar 1;50:9. doi: 10.1186/s40659-017-0111-1 (PMC5331689; doi:10.1186/s40659-017-0111-1)

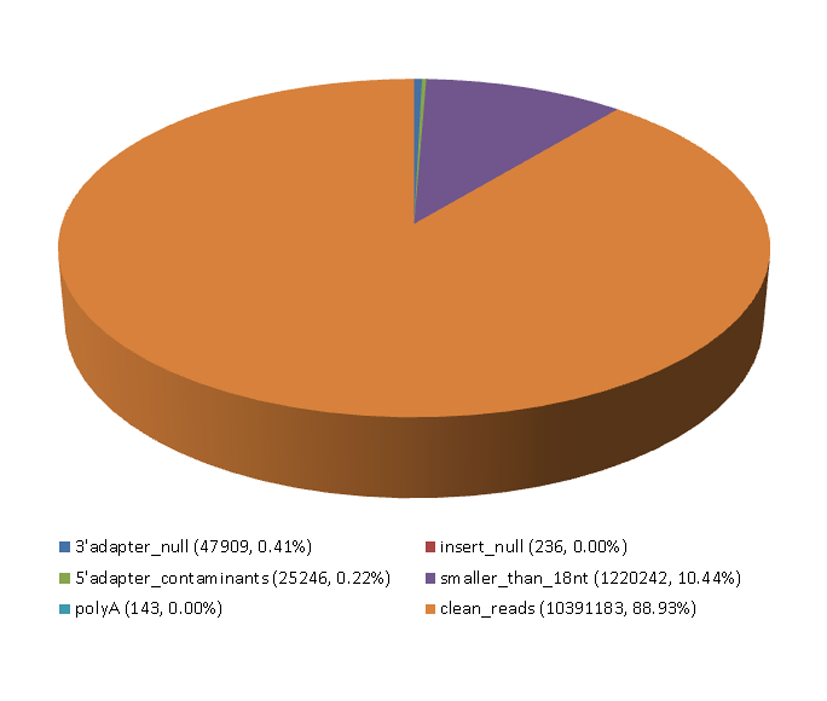

Supplement: Supplementary file 1 — Additional file 1. Overview of mouse transcriptome sequencing reads. [file 40659_2017_111_MOESM1_ESM.tif]

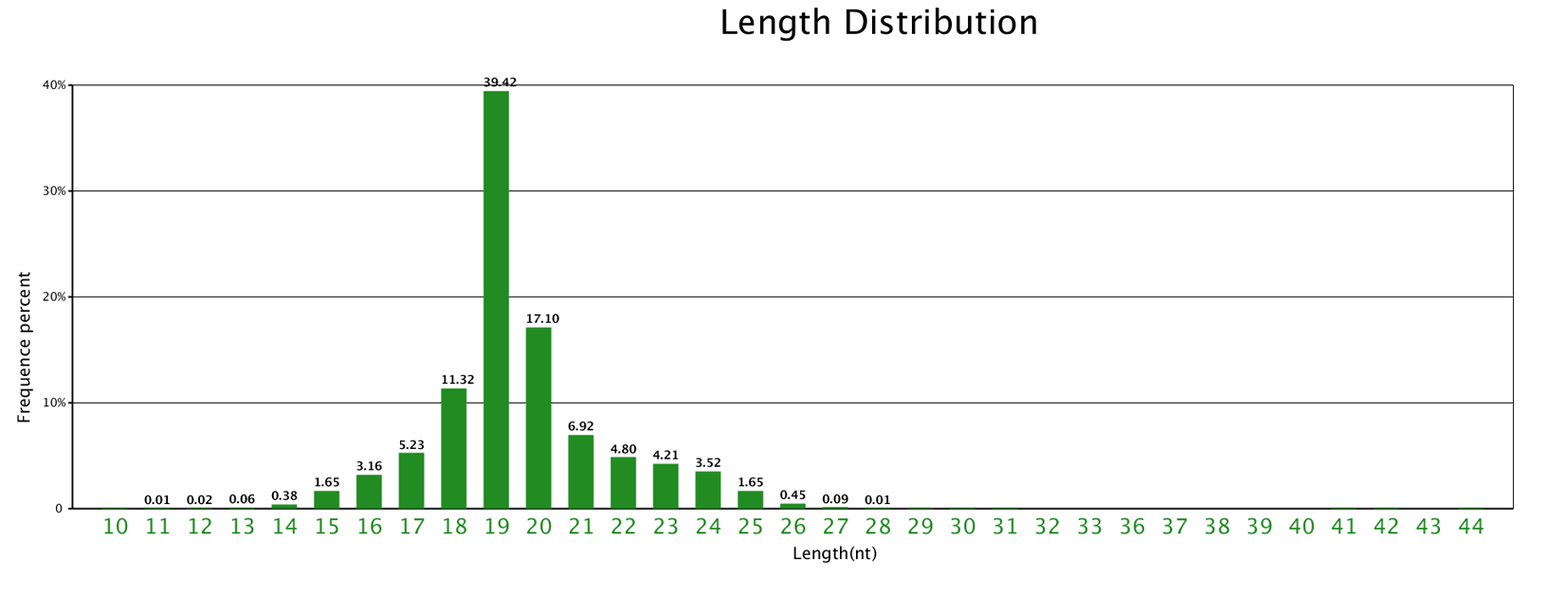

Supplement: Supplementary file 2 — Additional file 2. The sequence length distribution and frequence percentage of small RNA reads of mouse. The x-axis indicates the length of small RNA reads. The y-axis indicates the percentage of small RNA reads with specific length. [file 40659_2017_111_MOESM2_ESM.tif]
